# Correction: Preclinical amyloid pathology biomarker positivity: effects on tau pathology and neurodegeneration

**DOI:** 10.1038/s41398-018-0301-9

**Published:** 2018-11-19

**Authors:** K. Höglund, S. Kern, A. Zettergren, A. Börjesson-Hansson, H. Zetterberg, I. Skoog, K. Blennow

**Affiliations:** 1 Clinical Neurochemistry Laboratory, Institute of Neuroscience and Physiology, Department of Psychiatry and Neurochemistry, The Sahlgrenska Academy, Centre for ageing and Health, AgeCap, University of Gothenburg, Sahlgrenska University Hospital, Mölndal, Sweden; 2 Department of Neurobiology, Care Sciences and Society, Center for Alzheimer Disease Research, Neurogeriatrics Division, Karolinska Institutet, Novum, Stockholm Sweden; 3 Department of Molecular Neuroscience, UCL Institute of Neurology, Queen Square, London, UK; 4 Neuropsychiatric Epidemiology Unit, Institute of Neuroscience and Physiology, Department of Psychiatry and Neurochemistry, The Sahlgrenska Academy, Centre for Ageing and Health, AgeCap, University of Gothenburg, Mölndal, Sweden

**Correction to: Translational Psychiatry;**

10.1038/tp.2016.252; published online 10 January 2017.

The Article was originally published under a CC BY-NC-ND 4.0 license, but has now been made available under a CC BY 4.0 license. The PDF and HTML versions of the Article have not been corrected.

